# Failure to follow up on a medically actionable finding from direct to consumer genetic testing: A case report

**DOI:** 10.1002/mgg3.1252

**Published:** 2020-04-23

**Authors:** Ramin Garmany, Christopher J. Lee, Richard R. Sharp, Iftikhar J. Kullo

**Affiliations:** ^1^ Department of Cardiovascular Medicine Mayo Clinic Rochester MN USA; ^2^ Biomedical Ethics Research Program Mayo Clinic Rochester MN USA

**Keywords:** C282Y *HFE*, direct to consumer genetic testing, genetic counseling, hereditary hemochromatosis

## Abstract

**Background:**

A 61‐year‐old woman underwent direct to consumer genetic testing and was found to be homozygous for the C282Y *HFE* variant (c.845G>A :p.Cys282Tyr) which is classified as pathogenic/likely pathogenic for hereditary hemochromatosis. However, no action was taken by the individual.

**Methods:**

The individual took part in the Mayo Clinic Return of Actionable Variants Empiric (RAVE) study and the actionable finding was confirmed and results disclosed in person by a genetic counselor with subsequent referral to a hepatologist.

**Results:**

Further testing revealed iron overload with an elevated ferritin level (560 ng/ml) and increased ferritin saturation (74%). Phlebotomy was initiated with subsequent normalization of the ferritin levels (252 ng/ml).

**Conclusion:**

This case highlights that actionable genetic results may not be acted on after direct to consumer testing and the need for effective genetic counseling after such testing.

## Graphical abstract

We describe the case of a 61‐year‐old woman who was found to be homozygous for the C282Y *HFE* variant on direct to consumer genetic testing but only took action when the result was returned by a genetic counselor as part of a subsequent research study. This case highlights that actionable genetic results may not be acted on after direct to consumer testing and the need for effective genetic counseling after such testing.

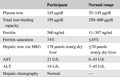

## 1 INTRODUCTION

Direct to consumer genetic testing (DTC‐GT) services are typically advertised as a means of exploring an individual's personal ancestry and/or providing insight into potential genetic predisposition to disease. The Food and Drug Administration (FDA) has reclassified DTC genetic tests as class II medical devices rather than class III medical devices subjecting them to a less stringent approval process (FDA, 2017). Consequently, this technology is likely to see an increase in commercial interest. In fact in 2017, the utilization of DTC genetic tests doubled (Regalado, 2018) and the market value of DTC genetic testing is expected to rise to $611 million by 2026 (Research, C, 2018). Recently, the FDA allowed 23andMe to provide testing for risk assessment of 10 heritable conditions (Allyse, Robinson, Ferber, & Sharp, 2018). Although DTC‐GT may empower consumers to take action based on genetic risk factors, it is unclear to what extent individuals follow up on actionable findings.

The Mayo Clinic Return of Actionable Variants Empiric (RAVE) study is a Mayo Clinic Institutional Review Board approved genomic medicine implementation study being conducted as part of the National Human Genome Research Institute (NHGRI)’s eMERGE network (Kullo et al., 2018). As part of this study, participants underwent sequencing of 68 actionable genes and 14 actionable single nucleotide variants. Those with a clinically actionable result were seen by a genetic counselor and referred to their primary care provider or a specialist as appropriate. Outcomes consequent to return of results were abstracted from the electronic health record. We present a case study of an individual who received an actionable finding on DTC‐GT but did not follow up on it until the same results were disclosed in person by a genetic counselor as part of the RAVE study. This report highlights the challenge of ensuring DTC‐GT consumers seek follow up on clinically actionable findings.

## 2 CLINICAL REPORT

A 61‐year‐old RAVE participant was found to be homozygous for the C282Y *HFE* variant (NM_000410.3(HFE):c.845G>A :p.Cys282Tyr) which is listed in ClinVar as pathogenic/likely pathogenic for hereditary hemochromatosis (HH). The participant did not carry a diagnosis of HH, although she had previously undergone DTC‐GT which revealed homozygosity for the *HFE* variant and also had a family history of the disease (a sibling had been diagnosed with HH and was receiving therapy). After DTC‐GT, the participant did not appear to have engaged with her physician to review this result.

After a genetic counselor disclosed the results as part of the RAVE study, the participant was referred to a hepatologist. Testing was consistent with HH and hepatic iron overload (Table 1). Weekly therapeutic phlebotomy was commenced and four months later the ferritin level had normalized (252 ng/ml).

## 3 DISCUSSION

DTC genetic testing arguably empowers consumers by providing access to some of their genomic information. Nonetheless, this case raises an important question whether individuals will follow up on actionable findings. Typically DTC‐GT companies provide either online or printed material to help consumers understand test results and also to provide information about when to engage with a physician. However, the information presented may not be sufficient for helping patients navigate their genetic testing results. In a 2008 survey of members of the National Society of Genetic Counselors, 65% believed the information provided as part of DTC‐GT was insufficient (Hock et al., 2011). For this reason, there have been calls for the companies to offer both pre‐test and post‐test genetic counseling for consumers (Harris, Kelly, & Wyatt, 2013).

Risk assessment using genetic information is complex since the role of genetics in disease is multifactorial and the estimates of penetrance for many variants are not reliable. Less than 10% of *HFE C282Y* homozygotes develop liver disease according to one study (Beutler, Felitti, Koziol, Ho, & Gelbart, 2002) while in another cross‐sectional study the penetrance in women was low ranging from 1%–14% (Rossi, Olynyk, & Jeffrey, 2008). The overall low and variable penetrance highlights the difficulty in decision making following receipt of genetic test results related to HH. The participant was aware that a first degree relative had a diagnosis of HH and required therapy and therefore had some insight into HH and its implications. Yet the participant did not follow up on the result until this was disclosed by a genetic counselor as part of the RAVE study.

This inaction has been previously alluded to in the literature. Bloss et al. (2013) demonstrated that DTC‐GT did not result in changes in health behaviors or screening unless the results were shared with a physician. This finding was also seen in individuals at increased risk for HH (Bloss et al., 2013). Only 40% of the participants in the study shared their results with a physician suggesting the presence of a “counseling gap” for DTC‐GT results which may be due to the requirement for counseling to be purchased separately or from an independent genetic counselor.

At present the onus remains on the consumer to place DTC‐GT results within their broader health goals and take action on the medically actionable results by pursuing follow up clinical interpretation or counseling. Within this context the role of physicians in helping patients follow up on DTC‐GT results is unclear. There is concern about the increasing workload of physicians in the primary care setting and that physicians may lack the training necessary to accurately counsel patients regarding DTC‐GT results (Covolo, Rubinelli, Ceretti, & Gelatti, 2015).

In the case presented, it is reasonable to assume that the in‐person genetic counseling session played a crucial role in triggering the appropriate medical care. Additional influences might have been participation in the study and subsequent interactions with the study team and referral to a specialist. Regardless, this case highlights the need for genetic counseling following receipt of medically actionable variants from DTC‐GT. Given the shortage of genetic counselors, innovative models of counseling individuals for such results from DTC‐GT need to be considered including using educational videos and online chat‐bots. Educating physicians about actionable findings from DTC‐GT may help ensure appropriate follow up medical care.

## CONFLICT OF INTEREST

None declared.

## AUTHORS’ CONTRIBUTIONS

C.J.L. and I.J.K. conceived and designed the study. R.G., C.J.L., and I.J.K. analyzed the data. R.G. and I.J.K. drafted the manuscript. All of the authors reviewed the manuscript.

4

**Table 1:** Baseline tests for iron overload and hepatic function

	Participant	Normal range
Plasma iron	145 µg/dl	35–145 µg/dl
Total iron binding capacity	195 µg/dl	250–400 µg/dl
Ferritin	560 ng/ml	11–307 ng/ml
Ferritin saturation	74%	≤45%
Hepatic iron (on MRI)	178 µmols iron/g dry liver	≤70 µmols iron/g dry liver
AST	21 U/L	8–43 U/L
ALT	19 U/L	7–45 U/L
Hepatic elastography	Normal	—
